# Glioma-derived extracellular vesicles as drivers of immunotherapeutic resistance: mechanisms of immune reprogramming and metabolic intervention

**DOI:** 10.3389/fimmu.2026.1859282

**Published:** 2026-05-22

**Authors:** Jiaxin Wang, Bo Peng, Ting Li, Tianduo Wang

**Affiliations:** 1 Affiliated Hospital of Liaoning University of Traditional Chinese Medicine, Shenyang, China; 2 Liaoning Cancer Hospital and Institute, Shenyang, China

**Keywords:** extracellular vesicles, glioma, immunotherapeutic resistance, PD-L1, tumor microenvironment

## Abstract

Gliomas, particularly glioblastoma (GBM), secrete abundant extracellular vesicles (EVs) that function as critical mediators of intercellular communication within the tumor microenvironment. These EVs carry diverse bioactive cargoes—including immune checkpoint proteins, non-coding RNAs, metabolites, and metabolic regulators—and can traverse the blood–brain barrier, potentially through endothelial uptake, transcytosis, and glioma-associated barrier remodeling, thereby linking local and systemic immune regulation. This review summarizes current knowledge regarding the mechanisms by which glioma-derived EVs contribute to immunotherapeutic resistance. EV-associated programmed death-ligand 1 (PD-L1) directly engages PD-1 on T cells and may exert broader and more sustained immunosuppressive effects than tumor cell membrane-bound PD-L1 by enabling distal immune modulation and multivalent inhibitory signaling. Glioma-derived EVs also promote myeloid-derived suppressor cell expansion, drive tumor-associated macrophage and microglial polarization toward immunosuppressive phenotypes, impair dendritic cell-mediated antigen presentation, and collectively reinforce T-cell dysfunction. In addition, EV-enriched non-coding RNAs, such as miR-25-3p, miR-3591-3p, and lncRNA H19, regulate PI3K–AKT–mTOR, JAK2/STAT3, and related pathways to promote myeloid reprogramming, immune escape, and temozolomide resistance. Importantly, EV-mediated temozolomide resistance and immunotherapeutic resistance may be interconnected through shared mechanisms involving non-coding RNA transfer, metabolic remodeling, inhibitory cytokine signaling, and myeloid-driven immune suppression. Furthermore, glioma EVs participate in tumor-specific immunometabolic regulation, including lipid, adenosine, amino acid, and glycolytic pathways, while exerting context-dependent effects on antigen presentation by either suppressing dendritic cell maturation or, under certain conditions, functioning as antigen carriers to stimulate antitumor immunity. Collectively, glioma-derived EVs act as central organizers of immune escape and therapeutic resistance. Targeting EV biogenesis, release, uptake, or specific immunosuppressive cargoes may provide promising opportunities to overcome resistance and improve immunotherapy for glioma. Future studies should focus on deciphering EV-mediated immune regulatory networks, identifying robust glioma-specific EV biomarkers, standardizing EV detection platforms, and developing precision EV-based therapeutic strategies.

## 1 Introduction

Gliomas, especially glioblastoma (GBM), are characterized by high invasiveness, therapeutic resistance, and profoundly immunosuppressive microenvironments (1, 2). Despite continuous advances in surgery, radiotherapy, chemotherapy, and immune checkpoint blockade (ICB), patient prognosis remains poor (3, 4). One of the major barriers to treatment is the development of immunotherapeutic resistance (5). In recent years, extracellular vesicles (EVs) have attracted increasing attention as critical mediators of intercellular communication involved in tumor immunotherapeutic resistance (6, 7).

EVs are a heterogeneous class of membrane-enclosed particles secreted by cells, including exosomes, microvesicles, and other vesicular subtypes. They carry proteins, lipids, and various nucleic acids—such as mRNAs, microRNAs (miRNAs), long non-coding RNAs (lncRNAs), and circular RNAs (circRNAs)—thus facilitating the transfer of biomolecules and information between cells and participating in diverse physiological and pathological processes (8). EVs derived from glioma cells exhibit notable characteristics: their secretion rate is markedly higher than that of normal cells, their molecular composition mirrors tumor-specific features, and they can cross the BBB to affect remote immune cells, consequently establishing immunosuppressive environments in both local and systemic compartments (7, 9, 10).

The mechanisms by which EVs mediate immunotherapeutic resistance are multifaceted (9, 11). On the one hand, EVs directly regulate the functions of immune cells such as T cells, MDSCs, and TAMs by delivering immune checkpoint molecules including PD-L1 and B7-H4, thereby inducing T-cell exhaustion and regulatory T cell (Treg) generation (6, 12, 13). In parallel, ncRNAs carried by EVs target critical signaling pathways, driving the polarization of immunosuppressive cell populations and boosting the production of inhibitory cytokines. Furthermore, EVs are involved in immunometabolic reprogramming and antigen presentation, thereby strengthening the tumor’s immune escape network (14, 15).

Given their central role in immunotherapeutic resistance, EVs have emerged as promising therapeutic targets and diagnostic biomarkers. Engineered EVs as drug delivery carriers, EV-based vaccine platforms, and EV-derived risk scoring models all show encouraging clinical potential (16, 17). This review centers on the biological properties of EVs, systematically examines their molecular mechanisms underlying immunotherapeutic resistance in glioma, and investigates EV-targeted therapeutic strategies. The goal is to establish a theoretical foundation for comprehending tumor immunotherapeutic resistance and for advancing novel immunotherapeutic approaches.

## 2 Biological characteristics of EVs and their secretion features in gliomas

### 2.1 Definition, biogenesis, and secretion mechanisms of EVs

EVs are heterogeneous membrane-bound vesicles secreted by cells into the extracellular space (18, 19). They include EV derived from multivesicular bodies (MVBs), microvesicles generated by outward budding of the plasma membrane, and other vesicular subtypes. EVs carry proteins, lipids, and nucleic acids and serve as important mediators of intercellular communication in both physiological and pathological conditions (20). Among these, EV typically range from 30 to 150 nm in diameter and derive from intracellular MVBs, whereas other EV subtypes vary in size, biogenesis route, and molecular makeup (21).

The generation of EVs relies on multiple pathways. The formation of EV entails the following steps: early endosome formation, MVB maturation, inward budding of the endosomal membrane to generate intraluminal vesicles (ILVs), and eventual fusion of MVBs with the plasma membrane to liberate EVs. This sequence is governed by both ESCRT-dependent and ESCRT-independent regulatory mechanisms (22, 23). In contrast, microvesicles arise from outward budding and fission of the plasma membrane, a mechanism that is tightly linked to cytoskeletal remodeling and lipid redistribution. Various regulatory molecules—including Rab GTPases, flotillin, and syntenin—participate in the biogenesis, cargo sorting, and release of EVs.

**Figure 1:**
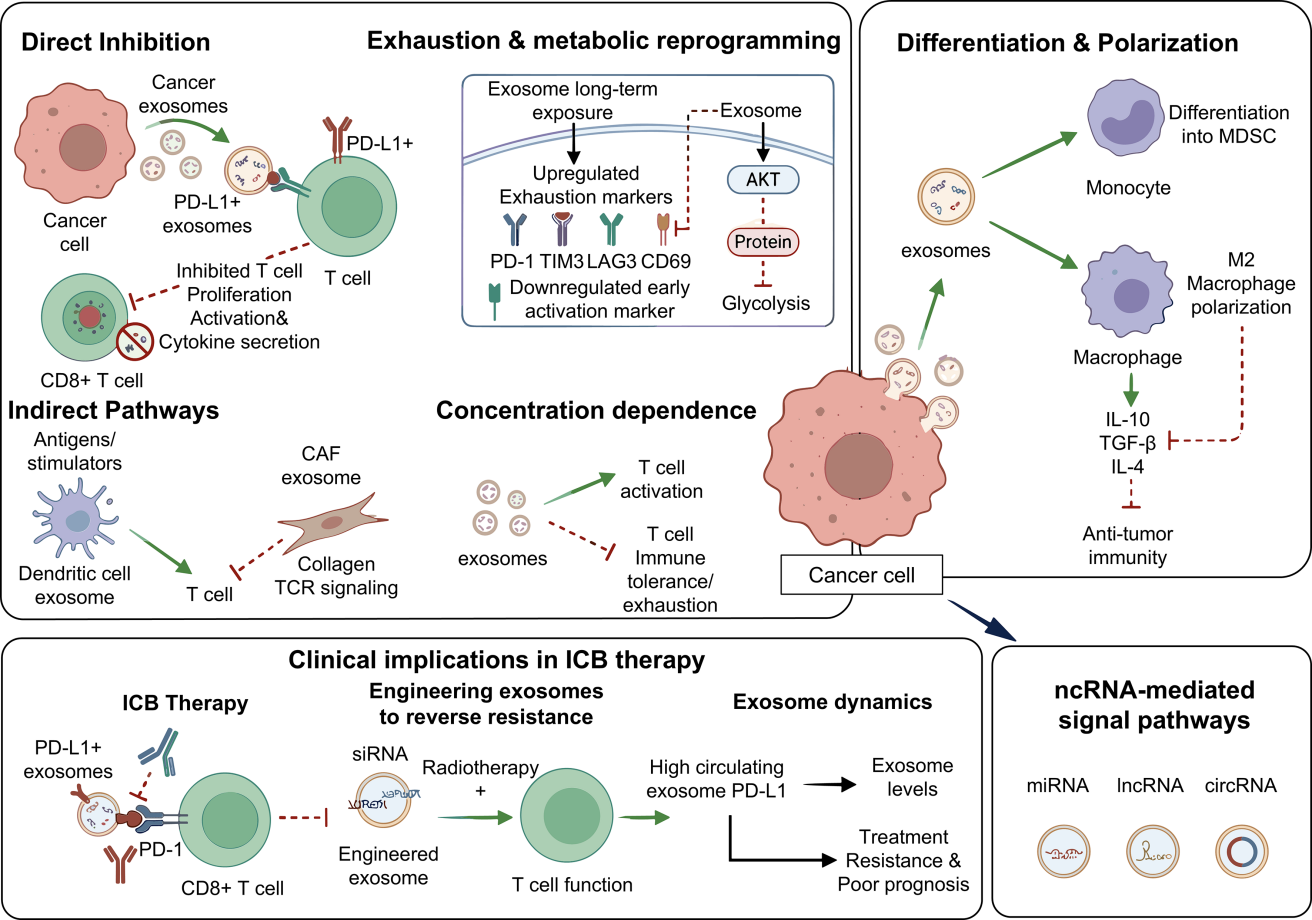
Biogenesis, cargo composition, and secretion pathways of EVs.

Multiple factors affect EV release, such as hypoxia, mechanical stress, autophagy, chemotherapy, and radiotherapy. Tumor cells secrete EVs at much higher levels than normal cells, and these vesicles are enriched with tumor-specific cargoes that mirror the molecular traits of their parent cells. These features underscore the essential roles of EVs in tumor progression, metastasis, immune escape, and resistance to immunotherapy (24–26) (**Figure 1**).

### 2.2 Specificity of glioma cell-derived EVs and their interactions with the tumor microenvironment

Gliomas release abundant EVs. These vesicles carry tumor-associated antigens, immunoregulatory factors, and ncRNAs, thereby mediating communication between tumors and the microenvironment (27–29). Glioma-derived EVs contain various oncogenic proteins and miRNAs that can regulate immune cell polarization and promote immune escape. For example, under hypoxic conditions, EV-associated lncRNA H19 is upregulated and induces microglial polarization toward a protumorigenic M2 phenotype while suppressing phagocytic function, thereby contributing to an immunosuppressive microenvironment (29). In addition, EV-associated miR-25-3p promotes macrophage M2 polarization through the PI3K–AKT–mTOR pathway, thereby accelerating tumor progression (30).

EVs can cross the BBB, enabling tumor-derived biomolecules to affect distant immune cells and modulate both central and peripheral immune responses (31). Mechanistically, EV passage across the BBB may involve several complementary routes. EVs can be internalized by brain microvascular endothelial cells through endocytosis-dependent pathways, including clathrin- or caveolin-mediated uptake and macropinocytosis, followed by transcytosis across the endothelial layer. In addition, interactions between EV surface molecules, such as integrins, tetraspanins, or other adhesion-related proteins, and endothelial receptors may facilitate selective binding and transport. In glioma, hypoxia, inflammation, VEGF-related signaling, and tumor-induced endothelial remodeling may further disrupt tight junction integrity and increase BBB or blood–tumor barrier permeability, thereby enhancing EV trafficking between the central nervous system and the peripheral immune compartment. By circulating through blood or cerebrospinal fluid, tumor-derived EVs shape a systemic immunosuppressive microenvironment and serve as bridges for dynamic interactions between tumor cells and immune/stromal cells, thereby regulating immune cell infiltration, polarization, and functional state, and ultimately affecting tumor growth, metastasis, and therapeutic resistance (32–34). Within the tumor microenvironment, EV-mediated intercellular communication is particularly important. Tumor-derived EVs interact with macrophages, microglia, stromal cells, and endothelial cells to form complex signaling networks (35, 36).

**Figure 2:**
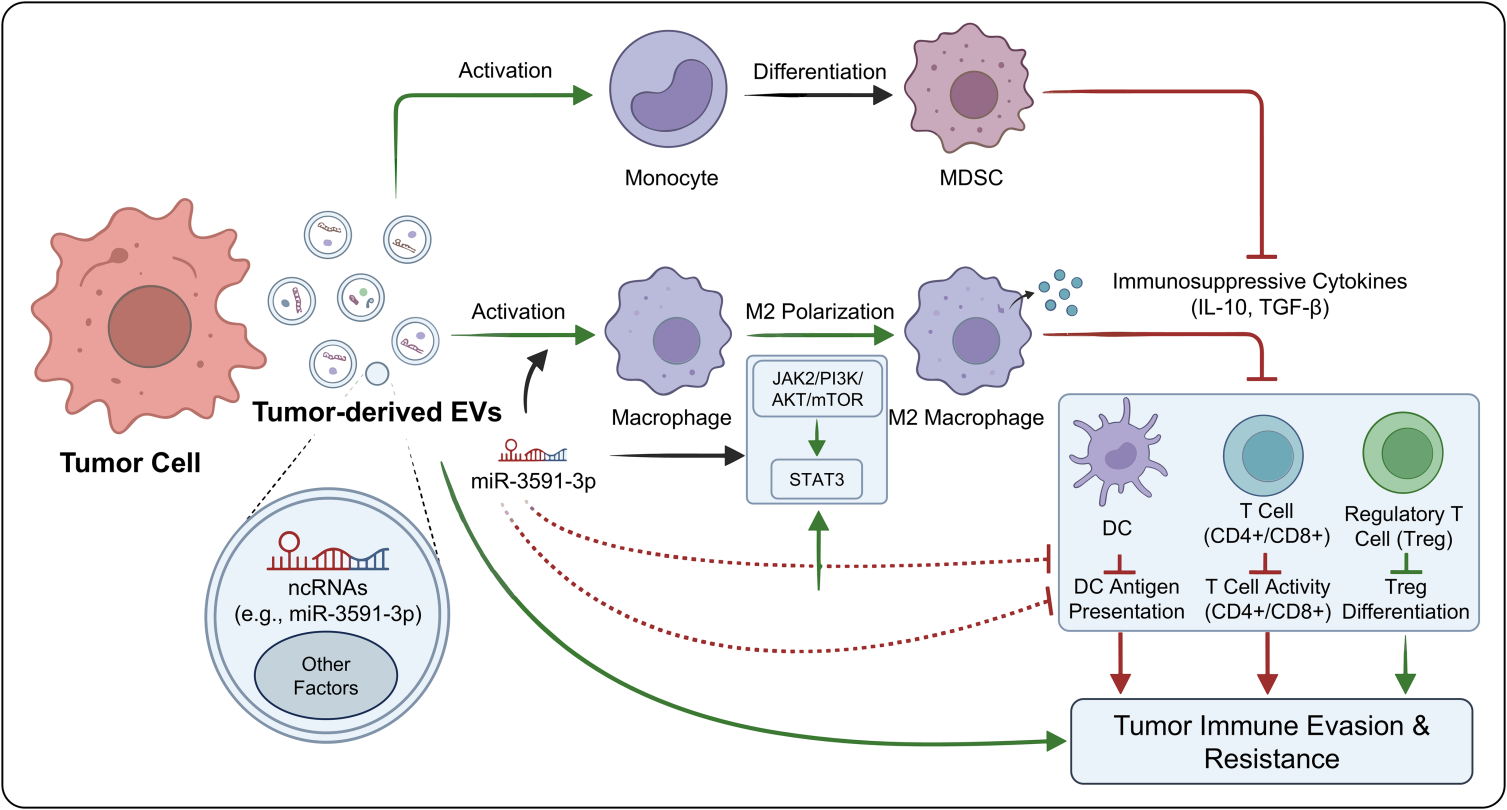
EVs mediate BBB crossing and dynamic tumor–microenvironment crosstalk.

Taken together, glioma-derived EVs are highly specific, carrying tumor-associated antigens, immunoregulatory factors, and ncRNAs. Their ability to cross the BBB and mediate dynamic communication between tumor cells and immune/stromal cells contributes to the establishment of an immunosuppressive microenvironment (37, 38). This mechanism deepens our understanding of CNS tumor immune escape and provides a theoretical basis for EV-based diagnostic and therapeutic strategies (**Figure 2**).

### 2.3 Overview of the role of EVs in tumor immunotherapeutic resistance

As key vehicles for intercellular communication, EVs exert multidimensional regulatory effects in tumor immunotherapeutic resistance (31). Tumor-derived EVs are enriched in bioactive molecules such as miRNAs, lncRNAs, and proteins that can remodel the immune microenvironment, regulate immune cell function, and promote immune escape and therapeutic resistance. For example, GBM-derived EVs deliver the immune checkpoint molecule B7-H4, thereby suppressing the antitumor activity of T cells and enhancing immune escape (12). Simultaneously, EVs can drive TAM polarization toward an immunosuppressive M2 phenotype, which fosters the establishment of an immunosuppressive microenvironment and subsequently promotes both immunotherapeutic resistance and tumor progression. These processes involve suppression of immune cells, regulation of immune checkpoints, and multiple layers of immune evasion, underscoring the multifaceted role of EVs in resistance to immunotherapy.

## 3 EV-Mediated regulation of immune cell function and mechanisms of immunotherapeutic resistance

### 3.1 Regulation of T-cell activation and function by EVs

Tumor-derived EVs play a critical role in the regulation of T-cell activation and function and represent a key component of tumor immune escape and therapeutic resistance (13, 39). Extensive evidence indicates that tumor cell-derived EVs carry multiple immunosuppressive molecules, among which PD-L1 is particularly prominent. By binding to programmed cell death protein 1 (PD-1) on T cells, PD-L1 directly suppresses T-cell activation and proliferation and weakens immune responses (11, 40).

The impact of EVs on T cells varies with concentration. At low doses, EVs can stimulate T cells, boosting their proliferation and activity; at high doses, however, they trigger immune tolerance or even exhaustion. Within the tumor setting, EVs likewise cause T-cell exhaustion, marked by elevated expression of checkpoint markers including PD-1, TIM3, and LAG3 along with diminished cytotoxic capacity, thus compromising the effectiveness of immunotherapy (40, 41).

**Figure 3:**
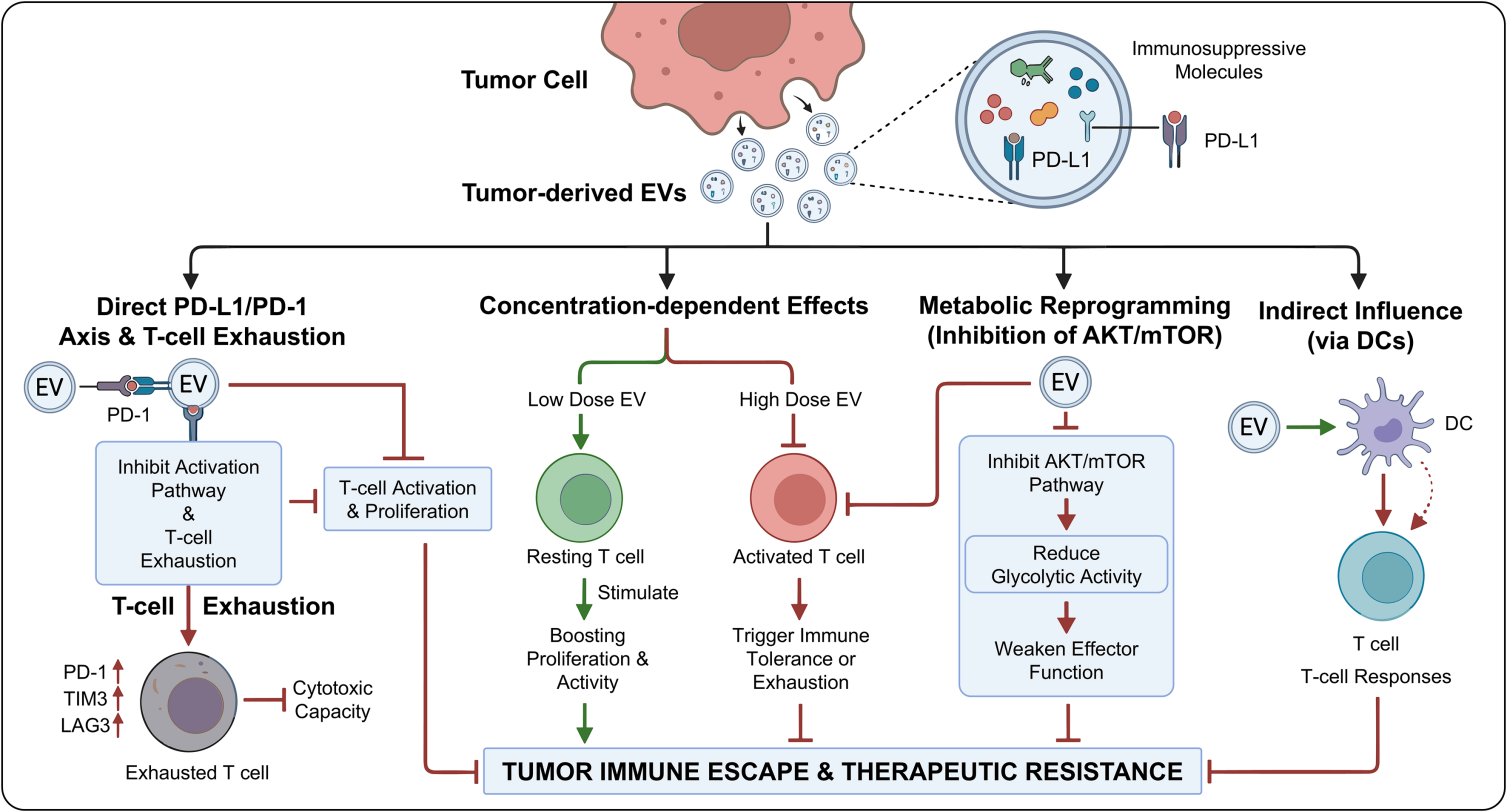
Regulation of T-cell activation and function by EVs.

In addition, EVs in the tumor microenvironment can indirectly influence T-cell responses by regulating antigen-presenting cells such as DCs. Tumor-derived EVs can also regulate T-cell metabolic reprogramming by inhibiting the AKT/mTOR pathway, reducing glycolytic activity, and weakening effector functions (40–42). Collectively, tumor-derived EVs profoundly influence T-cell activation and exhaustion through PD-L1-mediated signaling, concentration-dependent effects, and indirect pathways, making them major drivers of immunotherapeutic resistance (**Figure 3**).

### 3.2 EV-Induced differentiation of MDSCs and polarization of immunosuppressive macrophages

In gliomas, EVs play an active role in immune evasion and resistance by promoting MDSC differentiation and driving macrophage polarization. Tumor-derived EVs carry a range of immunoregulatory molecules—including miRNAs, lncRNAs, and proteins—that push monocytes toward an MDSC phenotype and strengthen their suppressive capacity. For example, EVs released by glioma cells overexpressing BATF2 suppress SDF-1α expression and reduce its presence within EVs, thereby disrupting the SDF-1α/CXCR4 signaling axis and diminishing the chemotaxis and recruitment of MDSCs. This suggests that tumor EVs not only boost the immunosuppressive activity of MDSCs but also regulate their accumulation in the microenvironment through chemotactic factor modulation, thereby building an immune barrier.

At the same time, tumor-derived EVs drive macrophages toward an M2 phenotype. M2 macrophages secrete immunosuppressive cytokines such as IL-10 and TGF-β, thereby promoting immune escape (31, 36). Glioma-derived EVs can also reprogram TAMs into tumor-supportive phenotypes while simultaneously suppressing DC antigen presentation, reducing CD4^+ and CD8^+ T-cell activity, and increasing the proportion of Tregs, resulting in comprehensive suppression of antitumor immunity (43).

**Figure 4:**
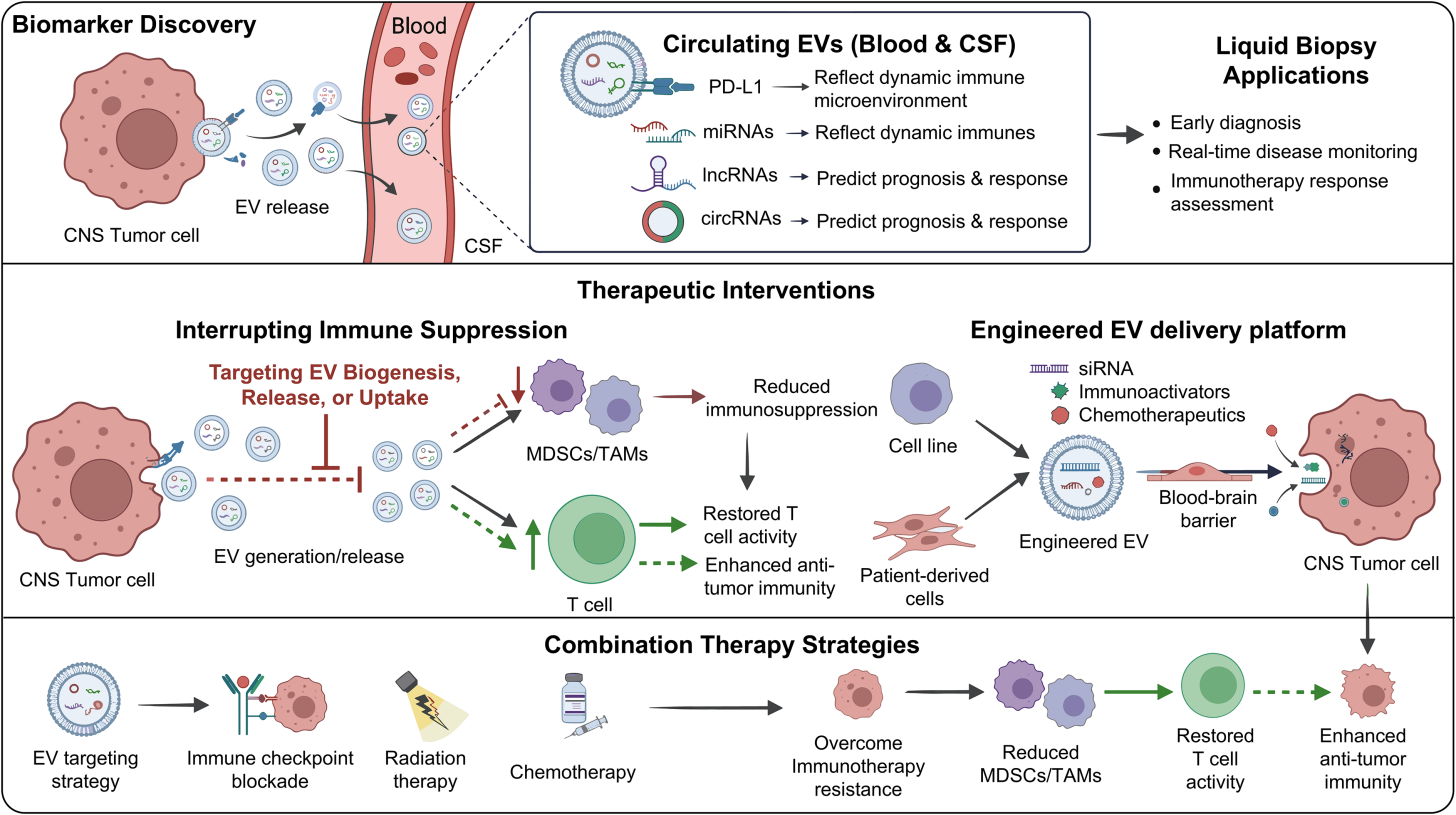
Regulation of MDSCs and M2 polarization by EVs.

At the molecular level, miRNAs carried by EVs play central regulatory roles. Glioma-derived EVs are enriched in miR-3591-3p, which promotes M2 polarization, increases IL-10 and TGF-β secretion, and activates the JAK2/PI3K/AKT/mTOR and STAT3 signaling pathways, thereby accelerating tumor progression (44). These findings underscore the central importance of EV-associated ncRNAs in immune cell reprogramming (**Figure 4**).

### 3.3 Regulation of immune checkpoint molecule expression by EVs

Immune checkpoint molecules present on EV surfaces, especially PD-L1, are key players in tumor immune evasion. EVs derived from tumors that carry PD-L1 engage with PD-1 on T cells, dampening T-cell effector functions and lowering cytotoxic activity, which in turn drives tumor progression. Hypoxic conditions further boost the release of PD-L1-enriched EVs from tumor cells, exacerbating immunosuppression and treatment resistance. Consequently, EV-associated PD-L1 serves not only as a crucial mediator of immune escape but also as a promising biomarker and therapeutic target (11, 40).

Compared with membrane-bound PD-L1 expressed on tumor cells, EV-associated PD-L1 may exhibit distinct functional properties. Tumor cell membrane PD-L1 mainly acts through direct cell–cell contact within the local tumor microenvironment, whereas EV-PD-L1 can diffuse or circulate to interact with T cells at both local and distant sites. Because multiple PD-L1 molecules can be displayed on the surface of a single EV, EV-PD-L1 may promote multivalent engagement with PD-1 and enhance inhibitory signaling. In addition, the relatively stable lipid bilayer of EVs may protect PD-L1 from rapid degradation, potentially prolonging its immunosuppressive activity. Therefore, EV-PD-L1 may contribute to broader and more sustained T-cell dysfunction than tumor cell membrane-bound PD-L1, although the exact differences in binding affinity, signaling duration, and exhaustion-inducing capacity require further quantitative investigation.

Apart from PD-L1, EV also affect the expression of other immune checkpoint molecules, further enhancing immune evasion. For instance, tumor-derived EV are rich in CTLA-4-related molecules, which inhibit the activation of CD4+ and CD8+ T cells by binding to ligands on antigen-presenting cells (45). EVs are also capable of transporting inhibitory cytokines like TGF-β, which modulate immune cell phenotype and function while fostering resistance to immune checkpoint blockade. Together, these varied mechanisms constitute an intricate network of immune evasion that significantly impacts the effectiveness of immunotherapy.

### 3.4 Roles of EV-carried non-coding RNAs in immunotherapeutic resistance

EVs are enriched in diverse ncRNAs, including miRNAs, lncRNAs, and circRNAs, which play crucial roles in tumor immunotherapeutic resistance (44). By secreting EVs containing specific ncRNAs, tumor cells regulate gene expression and functional states in immune cells, thereby promoting immune escape and resistance (30). EV-mediated ncRNA transfer not only affects tumor cells directly but also reshapes immune cell functions in the tumor microenvironment, thereby inducing immunosuppression and reducing the efficacy of immunotherapy (34) (**Table 1**).

**Table 1:** EV-mediated regulation of immune cell function and mechanisms of immunotherapeutic resistance in CNS tumors.

Regulatory aspect	EV source	Target cells/targets	Major effects and mechanisms of immunotherapeutic resistance
T-cell activation and functional regulation	Tumor-derived EVs carrying PD-L1	PD-1 on CD8^+ T cells	Inhibit T-cell activation, proliferation, and cytotoxicity; promote T-cell exhaustion with upregulation of PD-1, TIM3, and LAG3 (11, 40)
T-cell activation and functional regulation	High concentrations of tumor-derived EVs	T cells (concentration-dependent)	Low concentrations may activate T cells, whereas high concentrations induce immune tolerance or exhaustion and increase the proportion of Tregs (43)
T-cell activation and functional regulation	Cancer-associated fibroblast-derived EVs containing collagen	T-cell receptor signaling	Suppress T-cell receptor signaling and cause T-cell dysfunction
T-cell activation and functional regulation	Tumor-derived EVs	T-cell metabolism (AKT/mTOR)	Inhibit glycolytic activity and weaken effector function (43)
MDSCs and macrophage polarization	Glioma-derived EVs	MDSCs (CXCR4 signaling)	Block the SDF-1α/CXCR4 axis and reduce MDSC recruitment, while overall promoting MDSC immunosuppressive activity (46)
MDSCs and macrophage polarization	Medulloblastoma- and glioma-derived EVs	Macrophages/microglia	Induce M2 polarization and stimulate secretion of IL-10, TGF-β, and IL-4, thereby forming an immunosuppressive microenvironment (31)
MDSCs and macrophage polarization	Glioma-derived EVs	Macrophages (JAK2/PI3K/AKT/mTOR and STAT3 pathways)	Promote M2 polarization, increase IL-10 and TGF-β secretion, and accelerate tumor progression (30)
MDSCs and macrophage polarization	Glioma stem cell-derived EVs	Macrophages (miR-125a/STAT3 pathway)	Induce M2 macrophage polarization and reinforce the immunosuppressive microenvironment (3)
Regulation of immune checkpoint expression	Tumor-derived EVs	PD-1 on T cells	Systemically suppress antitumor immunity; hypoxia promotes PD-L1-bearing EV secretion and aggravates therapeutic resistance (11)
Regulation of immune checkpoint expression	Tumor-derived EVs	Ligands on antigen-presenting cells	Suppress activation of CD4^+ and CD8^+ T cells (47)
Regulation of immune checkpoint expression	Tumor-derived EVs	Immune cell phenotype and function	Promote resistance to ICB therapy (45)
Regulation of immune checkpoint expression	Engineered EVs delivering PD-L1 siRNA	PD-L1 expression in tumor cells	Reverse PD-L1 upregulation induced by radiotherapy, restore T-cell function, and overcome immunotherapeutic resistance (16)
ncRNA-mediated immunotherapeutic resistance	GBM-derived EVs	Microglia (C5/C5a)	Induce M2 polarization and promote TMZ resistance (28)
ncRNA-mediated immunotherapeutic resistance	Glioma-derived EVs	NCAM1 expression	Enhance glioma malignancy, migration, and sphere formation (43)
ncRNA-mediated immunotherapeutic resistance	Tumor-derived EVs	TAMs (ITGA5)	Stabilize ITGA5 protein, promote an immunosuppressive phenotype, and result in anti-PD-1 treatment failure (48)
ncRNA-mediated immunotherapeutic resistance	Glioma-derived EVs	miRNA target genes involved in DNA repair and metabolic pathways	Enhance TMZ resistance and promote tumor cell survival and immune escape (44)

Rather than acting on individual immune cell subsets in isolation, glioma-derived EVs may establish an interconnected immunosuppressive network among T cells, MDSCs, TAMs, and DCs. EV-mediated suppression of DC maturation and antigen presentation can reduce the priming and activation of effector T cells, while EV-induced MDSC expansion and TAM M2-like polarization further inhibit T-cell cytotoxicity through inhibitory cytokines, checkpoint molecules, and metabolic competition. In turn, dysfunctional T cells and immunosuppressive myeloid cells may reinforce each other by promoting Treg accumulation, sustaining signaling, and weakening antigen-specific immune recognition. Therefore, glioma-derived EVs should be viewed not only as regulators of single immune cell populations but also as organizers of a coordinated immune-suppressive circuit that links impaired antigen presentation, myeloid cell reprogramming, T-cell exhaustion, and immunotherapeutic resistance.

EV-mediated TMZ resistance and immunotherapeutic resistance may be closely interconnected rather than independent processes. TMZ treatment can reshape the molecular cargo and release pattern of glioma-derived EVs, thereby promoting the transfer of resistance-associated ncRNAs, DNA repair-related signals, metabolic regulators, and immunosuppressive molecules to tumor and immune cells. These EVs may enhance tumor cell survival while simultaneously remodeling the immune microenvironment by promoting M2-like polarization of macrophages/microglia, impairing dendritic cell-mediated antigen presentation, increasing inhibitory cytokine production, and weakening effector T-cell function. Conversely, an immunosuppressive microenvironment characterized by TAM accumulation, T-cell exhaustion, and elevated IL-10/TGF-β signaling may provide a protective niche that facilitates tumor cell persistence under TMZ pressure. Therefore, EVs may serve as a mechanistic bridge linking chemoresistance and immunoresistance in glioma, suggesting that combined strategies targeting EV release, cargo transfer, or EV-driven myeloid reprogramming may improve the efficacy of TMZ-based chemoimmunotherapy.

## 4 EVs and immune escape mechanisms in the tumor microenvironment

### 4.1 EVs promote the recruitment and activation of immunosuppressive cell populations

In the microenvironment of gliomas, EVs, as a crucial intercellular communication medium, significantly promote the recruitment and accumulation of regulatory T cells (Tregs) and myeloid-derived suppressor cells (MDSCs) (6). Glioma-derived EV are rich in various miRNAs, which, by regulating specific signaling pathways, drive the differentiation and activation of MDSCs, strengthening their immunosuppressive function. At the same time, miR-25-3p in EV can inhibit PHLPP2, activate the PI3K-AKT-mTOR pathway, and induce macrophages to polarize towards the M2 type, further creating an immunosuppressive microenvironment (30). The large aggregation of these immunosuppressive cells not only weakens the anti-tumor ability of effector T cells but also provides favorable soil for tumor immune escape.

In addition to promoting the recruitment of immunosuppressive cells, EV can also induce these cells to secrete inhibitory cytokines such as IL-10 and TGF-β, thereby further suppressing the functions of effector immune cells. For example, miR-3591-3p carried by tumor EV can drive macrophages to transform towards the M2 phenotype, enhance their secretion of IL-10 and TGF-β, and promote tumor invasion and migration (44). Under hypoxic conditions, lncRNA H19 in glioma EV can regulate microglia to transform towards a tumor-promoting M2 phenotype, inhibit their phagocytic activity, and jointly shape an immunosuppressive microenvironment (28). The release of these cytokines not only directly inhibits the activity of cytotoxic T cells and natural killer cells but also affects other cell types in the microenvironment, constructing a complex immune escape network.

Furthermore, EV-mediated immunosuppression also involves the regulation of tumor-associated macrophages (TAMs) and astrocytes (49–51). Glioma EVs can activate astrocytes, enhancing their autophagic activity and promoting the development of immunosuppressive phenotypes, which in turn supports tumor growth and metastasis. The molecular composition of EVs—including miRNAs and proteins—is closely associated with glioma resistance to temozolomide and the maintenance of an immunosuppressive state. Such multi-cellular, multi-pathway interactions create a profoundly immunosuppressive microenvironment that allows tumor cells to effectively escape host immune surveillance.

**Figure 5:**
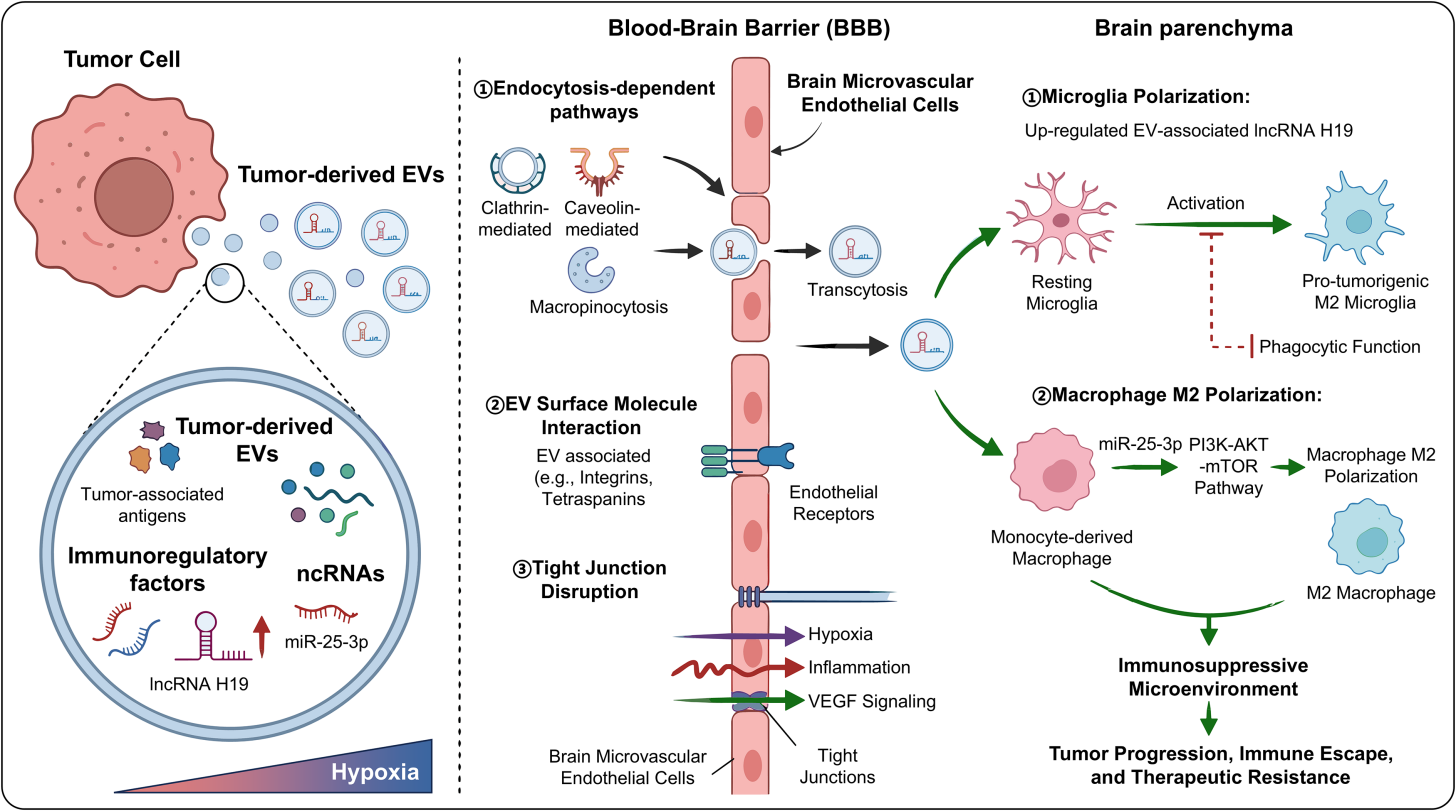
Mechanisms of extracellular vesicle-mediated immunotherapeutic resistance in CNS tumors.

In summary, gliomas release EVs that carry various immunomodulatory molecules, thereby facilitating the recruitment and activation of Tregs, MDSCs, and other suppressive cell subsets, while also stimulating the secretion of inhibitory cytokines. These processes collectively establish a complex immunosuppressive microenvironment. This environment not only compromises the anti-tumor activity of effector immune cells but also creates a favorable setting for tumor growth and treatment resistance. Future immunotherapeutic approaches may consider interfering with the delivery of key miRNAs or modulating EV release to overcome immune resistance and improve therapeutic outcomes (**Figure 5**).

### 4.2 EV-mediated immunometabolic regulation

EV can carry various bioactive molecules such as proteins, nucleic acids, and lipids, and participate in regulating the metabolic state of immune cells, thereby promoting the formation of immunosuppressive phenotype (52, 53)s. Within the tumor microenvironment, EVs can reshape the energy metabolism of immune cells by delivering specific metabolic enzymes, miRNAs, and other non-coding RNAs, thereby inducing immune tolerance and escape. In glioma, EV-mediated immunometabolic regulation may exhibit several tumor-type-specific features. Owing to the highly hypoxic, nutrient-restricted, and lipid-rich microenvironment of gliomas, glioma-derived EVs may preferentially remodel immune metabolism toward an immunosuppressive state. These EVs can affect macrophages, microglia, and T cells by transferring miRNAs, proteins, and metabolic regulators that converge on PI3K–AKT–mTOR, STAT3, lipid metabolism, and glycolysis-related pathways. As a result, glioma EVs may promote M2-like polarization of macrophages/microglia, weaken effector T-cell metabolic fitness, and support the accumulation of suppressive immune populations. Therefore, compared with EVs from peripheral tumors, glioma-derived EVs may have a more pronounced role in linking hypoxia-driven metabolic stress, myeloid cell reprogramming, and immune escape within the central nervous system microenvironment. Although evidence from other tumor types has provided important mechanistic insights, glioma-derived EVs should be interpreted within the unique metabolic and immune context of the CNS tumor microenvironment. For example, EVs secreted by hepatocellular carcinoma cells contain high levels of fatty acid binding protein 5 (FABP5). Once taken up by TAMs, FABP5 activates the PPARγ signaling pathway, leading to lipid accumulation and reprogrammed fatty acid metabolism. This drives macrophage polarization toward an immunosuppressive M2 phenotype, which in turn suppresses anti-tumor immune responses and accelerates tumor progression (14). This metabolic reprogramming not only alters the functional state of immune cells but also enhances the immunoreciprocity of the microenvironment by secreting immunosuppressive factors.

Adenosine metabolism regulation is another important mechanism mediated by EV in immune metabolism. Adenosine, as an immunosuppressive molecule in the tumor microenvironment, binds to the A2A receptor on immune cells, inhibiting the activity of effector T cells and natural killer cells, and promoting immune escape (15). EV can carry key enzymes or regulatory factors that regulate adenosine metabolism, enhancing adenosine production and its signal transduction, and exacerbating immunosuppression. For example, in immune thrombocytopenia, EV-mediated activation of the lectin complement pathway leads to metabolic disorders and functional impairment of immune cells, suggesting the crucial role of EV in regulating immune metabolism (43).

EVs also influence amino acid metabolism. Tumor cells and their EVs can impact the amino acid metabolic routes of immune cells—such as those involving glutamine and tryptophan—thereby altering the energy supply and signaling transduction of immune cells and sustaining an immunosuppressive phenotype. For example, EVs derived from TAMs are enriched in miRNAs that regulate oxidative stress and gluconeogenesis, which in turn modulate the metabolic adaptation of immune cells and facilitate tumor immune evasion. These metabolic adjustments place immune cells in a state characterized by low metabolic activity yet strong immunosuppression, a condition that favors tumor survival and progression.

**Figure 6:**
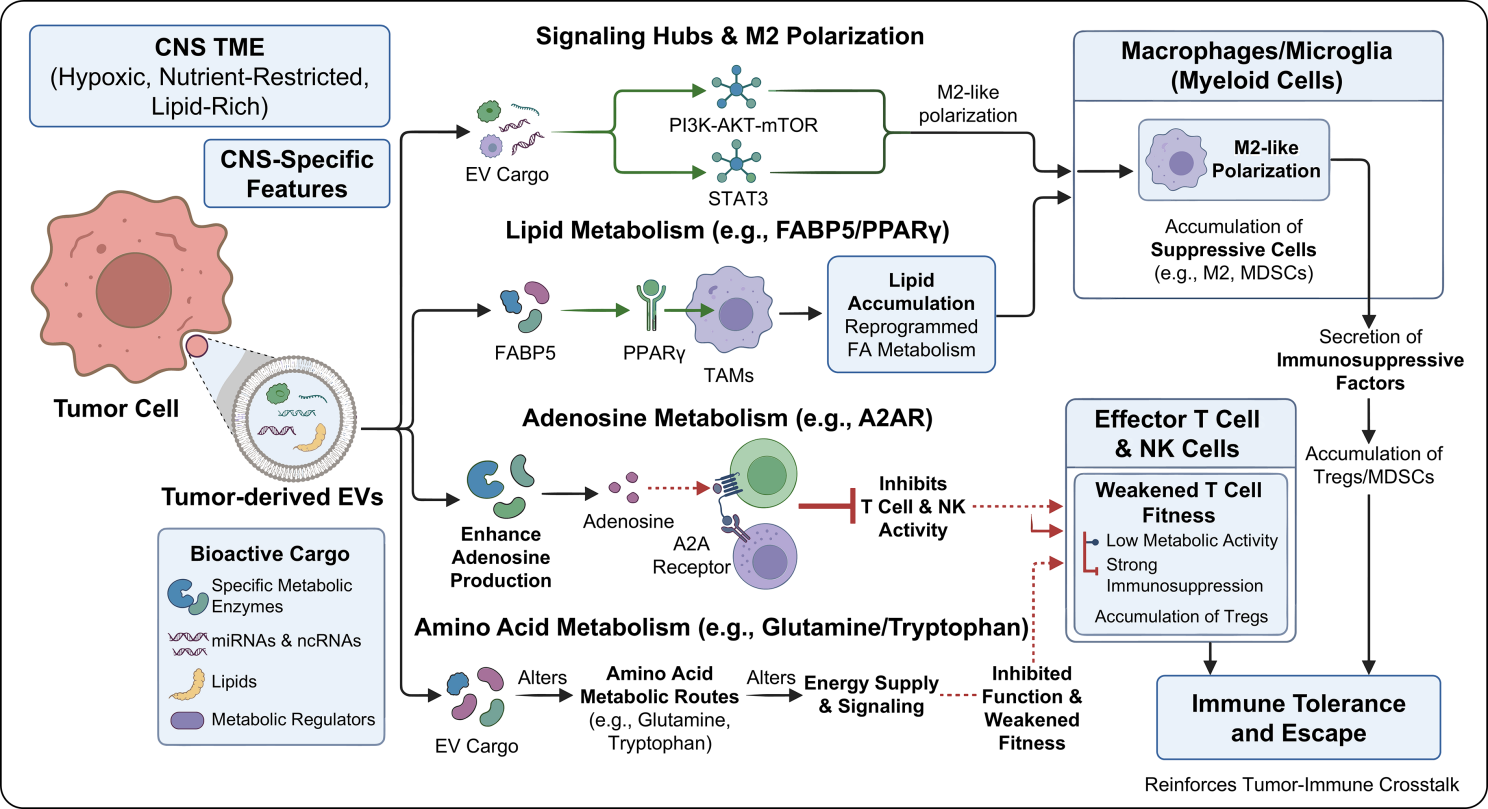
EV-mediated immunometabolic regulation.

In summary, EVs modulate the metabolic state of immune cells via multiple routes—including lipid, adenosine, and amino acid metabolism—thereby fostering the development of immunosuppressive phenotypes and reinforcing the immunoreciprocity of tumors. Targeting EV-mediated immune metabolic pathways represents a promising strategy to overcome tumor immune resistance. Of note, different tumor types and immune cell subsets exhibit varying responses to EV-driven metabolic regulation. Therefore, future investigations should thoroughly examine these mechanisms within the context of specific microenvironmental features to enable precision immunotherapy (**Figure 6**).

### 4.3 Interactions between EVs and tumor-associated macrophages

Tumor-associated macrophages are one of the most abundant immune cells in the tumor microenvironment and exhibit high plasticity and heterogeneity (45). EVs, as important mediators of intercellular signaling, carry active molecules such as proteins, nucleic acids, and lipids, mediating bidirectional communication between tumor cells and TAMs, regulating the polarization state and function of TAMs, and thereby influencing tumor immune evasion and progression (54). Studies have shown that glioblastoma cells secrete EV containing Podoplanin (PDPN), which are phagocytosed by bone marrow-derived macrophages. This significantly upregulates the expression of CD163 on the surface of macrophages, enhances the secretion of immune regulatory factors such as IL-6, IL-10, TNF-α, and TGF-β1, promotes M2 polarization, and simultaneously inhibits MHC II expression, weakening the activation ability of CD4+ T cells, forming an immune tolerance microenvironment (48). The immune inhibitory factors secreted by TAMs inhibit T cell activity, becoming a major obstacle to tumor immunotherapy.

In the tumor microenvironment, TAMs mainly present a M2 phenotype, with functions promoting tumor growth, promoting angiogenesis, and inhibiting immune responses. Tumor cells regulate the polarization of TAMs through non-coding RNAs (such as miRNA, circRNA) secreted by EVs, further promoting tumor progression. For example, miR-221-3p and let-7i-5p downregulated in EV derived from medulloblastoma can induce TAMs to polarize towards the M2-like phenotype by upregulating PPARγ, and PPARγ antagonists can enhance the anti-tumor activity of SMO inhibitors, suggesting their role in drug resistance (55). Similarly, EVcirc_0020256 secreted by TAMs in cholangiocarcinoma promote cancer cell proliferation and migration by regulating the miR-432-5p/E2F3 axis (56). These studies reveal that the molecular communication mediated by EV between tumors and TAMs is an important mechanism for regulating the immune microenvironment.

Conversely, EVs derived from M1-type macrophages can reprogram TAMs toward an M1-like phenotype, thereby exerting anti-tumor effects. Experimental evidence indicates that when IL-4-induced M2 macrophages are exposed to M1 macrophage EVs, they show marked upregulation of M1 markers (e.g., TNF-α), downregulation of M2 markers, and enhanced phagocytic capacity. Furthermore, engineered EVs designed to target the IL-4 receptor successfully converted M2-type TAMs into an M1-like state, leading to significant suppression of tumor growth and augmentation of anti-tumor immune responses. These findings highlight the therapeutic potential of modulating TAM polarization via EV-based strategies. However, it should also be noted that EVs secreted by TAMs themselves contribute to tumor immune evasion. TAM-derived EVs carry various miRNAs—including miR-155, miR-196a-5p, and miR-221-3p—which regulate tumor cell proliferation, migration, and immunosuppressive functions, promote treatment resistance through the PD-L1 pathway, mediate immune suppression, and ultimately worsen immune evasion (57). These findings indicate that TAMs and tumor cells form a complex immunoregulatory network through EV for bidirectional signaling.

In summary, tumor cells drive TAM polarization toward the M2 phenotype via EVs that carry molecules such as PDPN. In turn, M2-type TAMs release various immunosuppressive factors, which suppress effector T cell activity and thereby promote immune evasion and tumor progression. Modulating EV-mediated crosstalk between tumors and TAMs—particularly reversing the polarization state of TAMs—may represent an effective approach to overcome immune resistance. Future studies should further elucidate the EV-driven molecular mechanisms across different tumor types and assess their clinical translational potential, thereby offering new targets for immunotherapy.

### 4.4 EV-mediated regulation of tumor antigen presentation and immune recognition

EV play a crucial role in regulating tumor antigen presentation and immune recognition (58). EV secreted by tumor cells can directly reduce the recognition of tumors by the immune system by modulating the expression of major histocompatibility complex (MHC) molecules and the antigen-presenting ability. For instance, tumor EV in the cerebrospinal fluid of glioblastoma patients carry the specific protein LGALS9, which binds to the TIM3 receptor on dendritic cells (DCs), inhibiting the recognition, processing, and presentation of tumor antigens by DCs, leading to the failure of the cytotoxic T cell-mediated anti-tumor immune response (47). This indicates that tumor EV interfere with MHC-mediated antigen presentation, construct an immunosuppressive microenvironment, and promote immune escape.

The influence of EV on the function of dendritic cells is particularly crucial (59).

Dendritic cells (DCs) serve as a critical bridge between innate and adaptive immunity, and their maturation status and antigen-presenting capacity largely determine the strength of antitumor immune responses. Tumor-associated EVs (TAEs) can, under certain conditions, promote DC maturation and enhance MHC-mediated cross-presentation, thereby inducing tumor-specific cytotoxic T lymphocyte (CTL) responses. For example, TAEs derived from lung cancer cells have been shown to promote DC maturation more effectively than tumor cell lysates, reduce PD-L1 expression on DCs, decrease Treg abundance, and ultimately enhance antitumor immunity. These findings suggest that EVs exert context-dependent dual regulatory effects: while they may transmit immunosuppressive signals, they can also function as antigen carriers that stimulate antitumor immune responses. However, EV in the tumor microenvironment are mostly immunosuppressive (60). Tumor-derived EVs hinder the maturation and function of DCs, disrupt the antigen presentation process, and lower the efficiency of T cell activation. For instance, EVs found in the serum of acute myeloid leukemia patients can induce immune tolerance in DCs, thereby weakening anti-tumor immune responses. Of note, certain cancer treatment modalities—such as oncolytic virus therapy—can trigger the release of tumor EVs that, conversely, enhance DC maturation and activation. This suggests that the functional impact of EVs is shaped by both the tumor state and the treatment approach.

Dendritic cell-derived EVs (DEXs) possess distinct advantages for immunotherapy. EVs released by DCs can confer antigen-presenting capacity to monocytes, activate neoantigen-specific T cells, and amplify tumor-specific immune responses. For example, DC-derived EVs loaded with neoantigen peptides can promote the acquisition of DC-like features by monocytes, stimulate neoantigen-reactive T cells, and enhance antitumor immunity. This immune-amplifying effect provides a promising rationale for the development of EV-based tumor vaccines.

**Figure 7:**
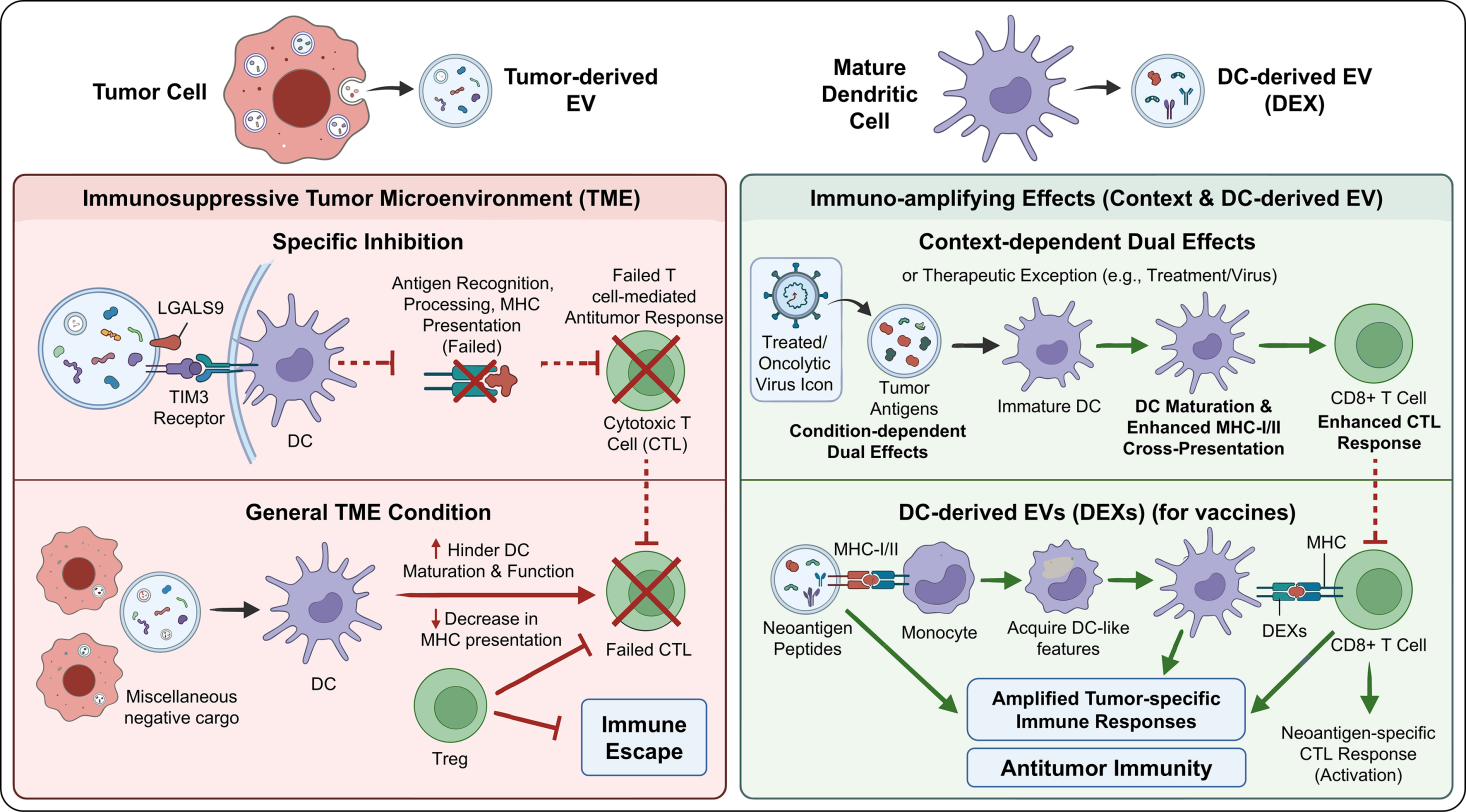
EV-mediated dual regulation of tumor antigen presentation and immune recognition.

**Figure 8:**
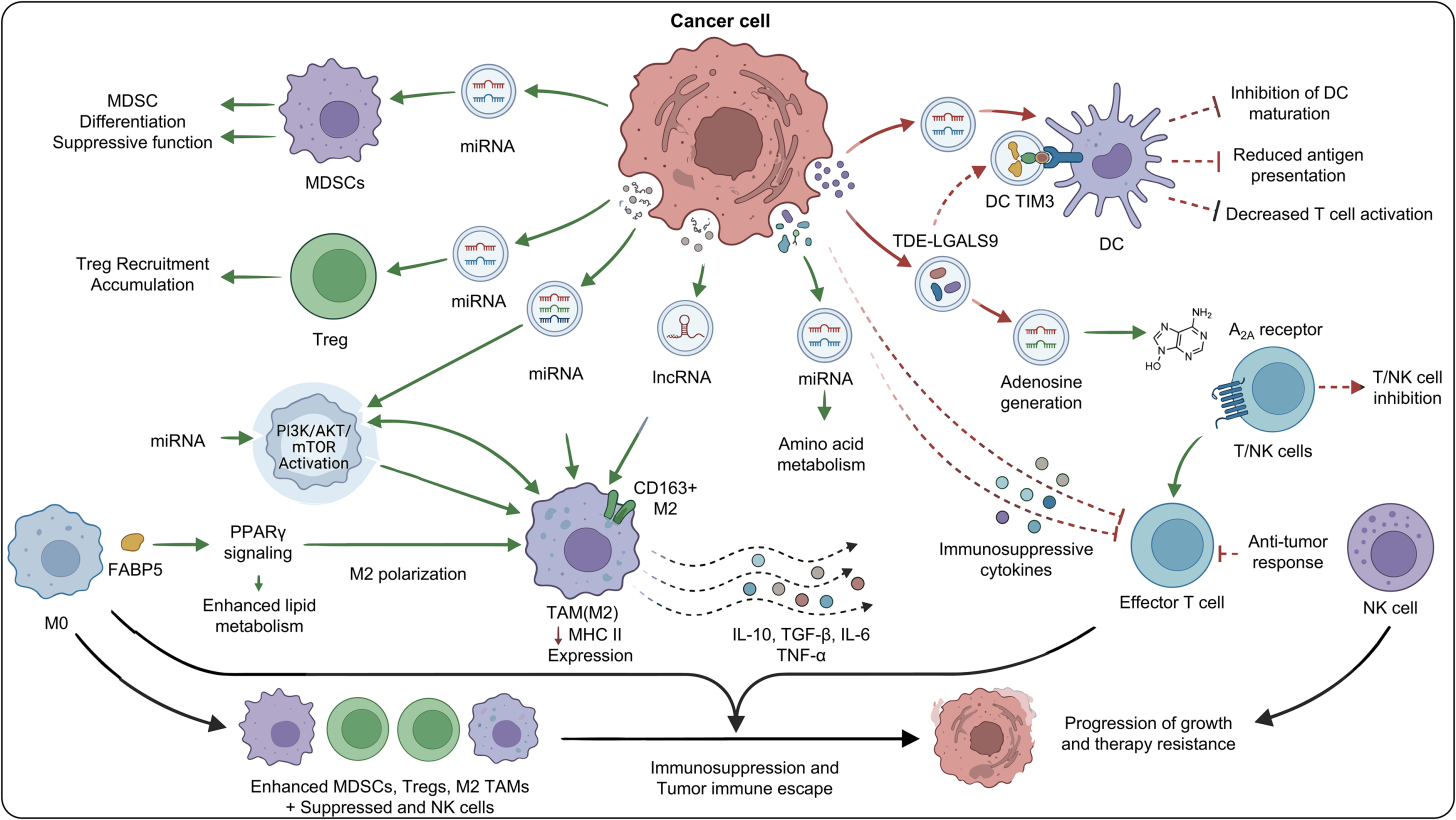
Overview of EV biomarker discovery, therapeutic interventions, and combination strategies in gliomas.

Overall, EVs substantially influence tumor immune recognition by regulating MHC molecule expression and antigen-presenting capacity. Tumor-derived EVs predominantly suppress DC function, thereby weakening the ability of the immune system to recognize and eliminate tumor cells. Conversely, under specific conditions, certain EV populations can promote DC maturation and enhance antitumor immune responses. Thus, DC-derived EVs may serve as potent activators of antitumor immunity and hold considerable translational potential. Targeting EV-mediated regulation of antigen presentation may therefore represent a promising strategy to overcome tumor immune resistance (**Table 2**; **Figures 7**, **8**).

**Table 2:** EV-mediated immune escape mechanisms in the tumor microenvironment of gliomas.

Mechanistic category	EV source	Target cells/pathways	Major immune escape effects
Promotion of suppressive cell recruitment and activation	Glioma-derived EVs	MDSCs	Drive MDSC differentiation and activation and enhance immunosuppressive function (1)
Promotion of suppressive cell recruitment and activation	Glioma-derived EVs	Macrophages (PI3K–AKT–mTOR pathway)	Induce M2 polarization and establish an immunosuppressive microenvironment (30)
Promotion of suppressive cell recruitment and activation	Glioma-derived EVs	Macrophages	Promote M2 polarization, increase IL-10 and TGF-β secretion, and suppress effector T cells and NK cells (10)
Promotion of suppressive cell recruitment and activation	Hypoxic glioma-derived EVs	Microglia	Induce M2 polarization and suppress phagocytic activity (28)
Promotion of suppressive cell recruitment and activation	Glioma-derived EVs	Astrocytes	Activate autophagy and immunosuppressive phenotypes, thereby supporting tumor growth and metastasis (32)
Immunometabolic regulation	Hepatocellular carcinoma-derived EVs	TAMs (PPARγ signaling)	Promote lipid accumulation and fatty acid metabolic reprogramming, driving M2 polarization (14)
Immunometabolic regulation	Tumor-derived EVs	Effector T cells, NK cells (A2A receptor)	Enhance adenosine production and suppress T-cell and NK-cell activity
Immunometabolic regulation	Tumor-derived EVs	Immune cells (amino acid metabolic pathways)	Alter energy supply and maintain immunosuppressive phenotypes (15)
Interactions with TAMs	GBM-derived EVs	Bone marrow-derived macrophages	Upregulate CD163, promote M2 polarization, suppress MHC II expression, and weaken CD4^+ T-cell activation (48)
Interactions with TAMs	Medulloblastoma-derived EVs	TAMs (upregulated PPARγ)	Induce M2-like polarization and promote therapeutic resistance (55)
Interactions with TAMs	M1 macrophage-derived EVs	M2-type TAMs	Reprogram TAMs to an M1-like phenotype, enhance phagocytosis, and exert antitumor effects
Interactions with TAMs	TAM-derived EVs	Tumor cells, PD-L1 expression	Promote tumor proliferation and migration and upregulate PD-L1-mediated immunosuppression (38)
Regulation of antigen presentation and immune recognition	GBM-derived EVs	DCs (TIM3 receptor)	Inhibit DC recognition, processing, and presentation of tumor antigens, leading to CTL failure (47)
Regulation of antigen presentation and immune recognition	Tumor-associated EVs	DCs	Promote DC maturation, enhance MHC cross-presentation, and activate CTLs (dual role) (39)
Regulation of antigen presentation and immune recognition	Tumor-derived EVs	DCs	Suppress DC maturation and function and induce immune tolerance
Regulation of antigen presentation and immune recognition	Dendritic cell-derived EVs	Monocytes, T cells	Induce antigen-presenting capacity in monocytes and activate neoantigen-specific T cells

## 5 Conclusion

As key mediators of communication between gliomas and the immune microenvironment, EVs play indispensable roles in the initiation and progression of immunotherapeutic resistance. This review systematically summarizes the biological properties and secretion characteristics of EVs in glioma, with particular emphasis on the mechanisms by which they promote resistance to immunotherapy. Glioma-derived EVs transport immune checkpoint molecules, deliver ncRNAs, reshape immunometabolic programs, and impair DC-mediated antigen presentation, thereby facilitating immune escape and therapeutic resistance. Moreover, their ability to cross the BBB enables them to act as critical connectors between the central nervous system tumor microenvironment and peripheral immune compartments.

Importantly, EVs exert dual and context-dependent effects on immune regulation. Although tumor-derived EVs are predominantly immunosuppressive, EVs originating from DCs or M1 macrophages can enhance antitumor immune responses. This functional duality provides a theoretical foundation for the development of precise EV-based immunotherapeutic strategies. Among EV-associated molecules, PD-L1 has emerged as a promising predictive biomarker for immunotherapy response, while engineered EV-based drug delivery systems and EV vaccine platforms have shown encouraging preclinical potential.

In summary, EVs represent central mediators of immunotherapeutic resistance in glioma. A deeper understanding of their molecular mechanisms is essential for overcoming immune evasion and improving patient outcomes. Future studies should prioritize the establishment of standardized protocols for EV isolation and characterization, the identification of robust tumor-specific EV biomarkers, the development of targeted interventions that modulate EV biogenesis, release, uptake, or cargo packaging, and the clinical translation of engineered EV-based therapeutic platforms.

The greatest technical barrier currently preventing glioma EV biomarkers from entering standard clinical testing is the lack of standardized and reproducible workflows for EV isolation, tumor-specific EV enrichment, and quantitative detection. In clinical samples, glioma-derived EVs represent only a small fraction of total circulating EVs and are mixed with vesicles released by normal blood cells, endothelial cells, immune cells, and other tissues. This heterogeneity makes it difficult to distinguish true tumor-derived EV signals from background vesicle populations. Moreover, differences in sample source, pre-analytical handling, isolation methods, marker selection, and detection platforms can lead to substantial inter-study variability. Therefore, future clinical translation will require standardized protocols, robust tumor-specific EV markers, sensitive detection technologies, and large multicenter validation cohorts.
